# Comparison of Postoperative Analgesic Effect of Dexamethasone and Fentanyl Added to Lidocaine through Axillary Block in Forearm Fracture

**DOI:** 10.1155/2013/761583

**Published:** 2013-12-29

**Authors:** Siamak Yaghoobi, Mahyar Seddighi, Zohreh Yazdi, Razieh Ghafouri, Marzieh Beigom Khezri

**Affiliations:** ^1^Department of Anesthesiology, Faculty of Medicine, Qazvin University of Medical Sciences, Shahid Bahonar Boulevard, Qazvin 3419759811, Iran; ^2^Department of Anesthesiology, Faculty of Medicine, Qazvin University of Medical Sciences, Iran; ^3^School of Medicine, Qazvin University of Medical Sciences, Qazvin, Iran

## Abstract

*Aim*. Regional analgesia has been introduced as better analgesic technique compared to using systemic analgesic agents, and it may decrease the adverse effects of them and increase the degree of satisfaction. Several additives have been suggested to enhance analgesic effect of local anesthetic agents such as opioids and steroids. We designed this randomized double-blind controlled study to compare the analgesic efficacy of the dexamethasone and fentanyl added to lidocaine using axillary block in patients undergoing operation of forearm fracture. *Materials and Methods*. Seventy-eight patients 20–60 years old were recruited in a prospective, double-blinded, randomized way. Axillary block was performed in the three groups by using 40 mL lidocaine and 2 mL distilled water (L group), 40 mL lidocaine and 2 mL dexamethasone (LD group), and 40 mL lidocaine and 2 mL fentanyl (LF group). The onset time of sensory and motor block, duration of sensory and motor block, the total analgesic dose administered during 6 hours after the surgery, and hemodynamic variables were recorded. *Results*. The duration of sensory and motor block was significantly longer in LD group compared to other groups (*P* < 0.001). Similarly, the total analgesic consumption in LD group was smaller compared to other groups (*P* < 0.001). Comparison of hemodynamic consequences of axillary block and surgery failed to reveal any statistically significant differences between all groups. *Conclusion*. Addition of dexamethasone to lidocaine significantly prolonged the duration of analgesia compared with fentanyl/lidocaine mixture or lidocaine alone using axillary block in patients undergoing forearm fracture surgery. This trial is registered with IRCT2012120711687N1.

## 1. Introduction

Postoperative pain is associated with neuroendocrine responses, catecholamine release, and increased morbidity and the central sensitization is believed to be among the mechanisms implicated in the persistence of postoperative pain [1].

Regional techniques have been suggested to produce superior analgesia, compared to systemic opioids, and they may even improve the final outcomes and also decrease the adverse effects of narcotics and increase the degree of satisfaction [2].

There are many additives to be used to enhance analgesic effect of regional block such as clonidine, magnesium, opioids, vasoconstrictor agents, and steroids [3, 4].

Opioids are considered as cornerstone for treatment of pain following surgery. Furthermore, it is reported that opioid antinociception can be initiated by activation of peripheral opioid receptors [5]. The presence of peripheral opioid receptors is shown in immune cells and primary afferent neurons in animals and also on the peripheral terminals of primary afferent nerves [6–8]. Furthermore, it is reported that fentanyl has a local anesthetic action [9].

The addition of opioids to local anesthetics injected into brachial plexus network was shown to increase the success rate and postoperation analgesia by some authors [10–13], whereas others have found no effect [14, 15].

Nevertheless, it is reported that a single administration of an opioid may also induce a long-lasting increase of threshold pain sensitivity, leading to delayed hyperalgesia [16].

On the contrary, it is reported that dexamethasone increases the regional block-associated analgesia due to either inhibitory activity of potassium channels on pain-sensitive sensory neurons or production of some extent of vasoconstriction [17]. The use of dexamethasone as an adjuvant to local anesthetic has been the subject of previous investigations [17–20], but to the best of our knowledge, the present research is the first study in which the analgesic effect of dexamethasone was compared to fentanyl.

We hypothesized that dexamethasone may provide a better pain relief after orthopedic surgery under axillary block compared to fentanyl. In addition, unlike opioids, dexamethasone does not produce pruritus, respiratory depression, or hyperalgesia. To test our hypothesis, we designed this randomized double-blind, placebo-controlled study to compare the postoperative analgesic effect and the success rate of axillary block while dexamethasone or fentanyl was added to lidocaine in patients undergoing forearm surgery.

## 2. Methods

**Figure 1:**
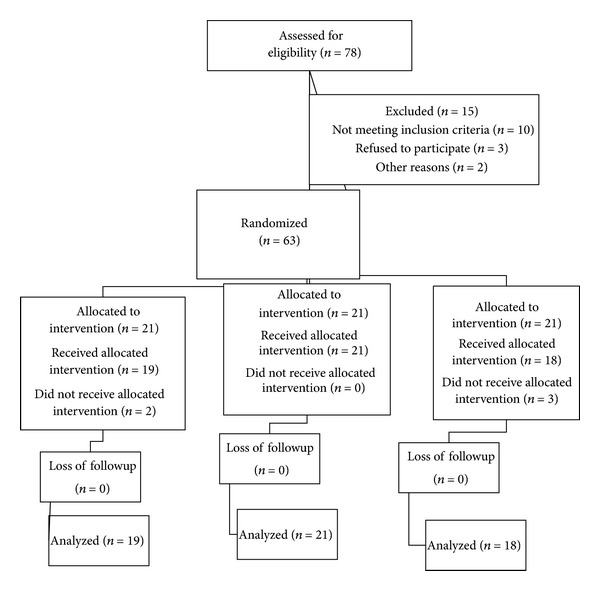
CONSORT flow of diagram.

Following the approval by the Ethics Committee of Medical School, Qazvin University of Medical Sciences, and obtaining informed patient consent, seventy-eight patients 20–60 years old, with American Society of Anesthesiologists (ASA) physical status I or II, scheduled elective open reduction forearm fracture surgery under axillary block, were recruited in a prospective, double-blinded, randomized way. The Consolidated Standards of Reporting Trials (CONSORT) recommendations for reporting the randomized, controlled clinical trials [21] were followed (Figure 1). The following inclusion criteria were considered: (a) presence of forearm fracture, (b) performance of open reduction surgery at Rajaei Hospital, (c) absence of underlying diseases such as coagulopathy, peripheral vascular diseases, and neuropathy, (d) patient's full agreement on performing axillary anesthesia, (e) lack of any history of allergy to local anesthetics, (f) lack of treatment with palliative drugs and those affecting the CNS, (g) being a nonaddict, and (h) suitability of patients to be placed (classified) in ASA I and II classes. Also, the exclusion criteria employed were (a) operation duration of more than 90 min and (b) the appearance of pain during the surgery. Patients were divided into 3 groups based on the balanced block randomization as follows: (L) lidocaine 1% (40 mL) + distilled water (2 mL), (LD) lidocaine 1% (40 mL) + dexamethasone (2 mL), and (LF) lidocaine 1% (40 mL) + fentanyl 100 *μ*g (2 mL). The 3 study groups were further marked as 6 blocks (ABC, ACB, BAC, BCA, CAB, and CBA) based on A, B, and C alphabets and numbers from 1 to 6. In the final stage, based on the random number table, blocks were arranged next to each other. The ward secretary, anesthetist, surgeon, and statistician were all unaware of blocks arrangement while the A, B, and C alphabets were placed in envelops and opened in the operation room before the performance of axillary block. There were 26 patients in each group.

After administration of 1 mg midazolam as premedication, axillary block was performed by transarterial technique. During the operative period, other sedative or analgesic agents were not administered. The intensity of pain was measured according to the visual analogue scales (VAS), graded from no pain (zero) on the left and the worst imaginable degree of pain (10) on the right. The injection time, blood pressure, and pulse rate were recorded. Following injection, the blood pressure and heart rate (HR) were measured every 5 min for a total of 20 min. The onset of sensory and motor block was evaluated by the pin-prick and the gripping force, respectively, at 30-seconds intervals. After the surgery, the return of sensory block was evaluated by pinprick technique and that of the motor block by the gripping force. The pain intensity with VAS, for a total duration of 6 hrs and at 2 hrs interval was evaluated. After the surgery, patients were requested to choose a point on the scale depending on the intensity of pain and in case of an intensity higher than 4 (based on VAS), routine analgesic (pethidine 25 mg) was administered. Eventually, the time for the commencement of pain, the quantity of VAS at the beginning of pain, and the total analgesic dose administered during 6 hrs after the surgery were recorded. The time of onset of sensory block was defined as the interval between the time of end of injection and loss of pain in pinprick test at around the injury site of forearm. The duration of sensory and motor block, respectively, was taken as the time interval between the time of loss of pain sensation at forearm and reappearance of paresthesia or complete recovery of motor function. The duration of analgesia was defined as the period from drug injection to the first occasion when the patient complained of pain. The duration of analgesia was the primary outcome variable on which sample size estimation was based. To calculate the sample size, data from previous similar studies were taken into consideration [12–15]. Sample size analysis determined that a total sample size of 75 (25 patients in each group) could make a sufficient power (90%) at a level of significance equal to 0.05 to detect 10% effect of intervention. Data was analyzed with SPSS16; ANOVA was employed to compare the demographic and hemodynamic variables to determine the effect of random allocation in 3 groups. Also, the differences in the results associated with the onset motor block, anesthesia, and the duration of analgesia between the three groups were compared using ANOVA. To determine the differences between two groups, the Tukey post hoc test was employed.

## 3. Results

Among 78 patients initially enrolled in this study, 15 patients had to be excluded because of logistical reasons or violations of the study protocol. Sixty-three patients were included and randomly assigned to the treatment groups. A total of 58 patients, allocated to 3 groups marked as L (19), LD (21), and LF (18), received intervention and the data were further analyzed by descriptive and analytical statistics.

As shown in Table 1, there was no significant difference associated with the age and gender between the 3 groups (*P* = 0.73).

Table 2 shows a significant difference in duration of sensory block between groups LD and LF (*P* < 0.001) and L (*P* < 0.001). Also, Table 2 is indicative of a significant difference in mean duration of motor blockade time between groups LD and LF (*P* < 0.001) and L (*P* < 0.001). Patients who were given combined lidocaine-dexamethasone demonstrated a significantly prolonged duration of analgesia compared with the lidocaine alone (*P* < 0.001) and fentanyl-lidocaine mixture groups (*P* < 0.022). As shown in Table 2, the difference in mean onset time of sensory block (0.44) and motor block (*P* = 0.68) between three groups was insignificant. As demonstrated in Table 2, there were significant differences among three groups associated with the analgesic doses administered and VAS (at time of return sensation).

As shown in Table 3, the hemodynamic variables including systolic and diastolic blood pressure and pulse rate at 0, 5, 10, 15, and 20 min after injection showed insignificant differences between the three study groups. No patient in either group had compartment syndrome or any complications related to anesthetic technique such as sensory or motor complications.

## 4. Discussion

The results found in the present study showed that addition of dexamethasone to lidocaine provided longer duration of analgesia compared to fentanyl/lidocaine mixture or lidocaine alone using axillary block in patients with forearm fracture surgery. Furthermore, patients experienced pain at lower intensity following the operation compared to the other two groups. Moreover, the analgesic dose administered for the group that received dexamethasone after the surgery was significantly lower than that used in the other two groups.

More recent publications since the aforementioned review indicate that 8 mg dexamethasone added to perineural local anesthetic injections augments the duration of peripheral nerve block analgesia [17–22]. On the contrary, it is reported that, in rats, dexamethasone alone or when combined with aqueous bupivacaine has no effect on the analgesic effects of a sciatic nerve block, but when combined with bupivacaine microspheres, the effects were significant [2, 23].

Shrestha and coworkers reported that addition of dexamethasone for brachial plexus block significantly prolongs the duration of analgesia without any unwanted effects [22].

In a study by Vieira et al., it was also shown that the addition of dexamethasone to bupivacaine increases the duration of analgesia, but no further evaluation on the intensity of pain and analgesic dose after the surgery was made [19]. In a paper by Movafegh et al., the addition of dexamethasone to lidocaine produced similar results on the duration of analgesia and motor block, but no data regarding the pain intensity and analgesic use following the operation were presented. Meanwhile, the addition of dexamethasone to lidocaine failed to decrease the time for the onset of sensory and motor block while the hemodynamic changes during the surgery were also described to be comparable with those of control group [24], a finding in agreement with our results.

There are several theories to explain this effect of dexamethasone; among those is the one representing on degree of vasoconstriction produced by steroids and this in turn decreases the absorption of local anesthetics [2, 17]. Secondly, theory indicates that dexamethasone increases the inhibitory activity of potassium channels on pain sensory nerves [2, 17]. Another theory refers to anti-inflammatory action of dexamethasone and blocking transmission of nociceptive C-fibers [2, 17].

In the present study, we showed that the addition of fentanyl to lidocaine significantly increased the duration of analgesia and motor block compared to the group given lidocaine alone; however, this drug combination failed to decrease the intensity of pain and analgesic dose, after operation. Furthermore, the time course for the onset of sensory and motor blocks remained unchanged and the hemodynamic changes were found to be similar to those observed for the lidocaine group. In a study by Nishikawa et al., it was also observed that the addition of fentanyl to lidocaine, similar to our study, increased the duration of analgesia and improved the success rate of sensory blockade [12]. Similarly, Karakaya et al. reported an increase in the duration of anesthesia and analgesia in axillary block following addition of fentanyl to bupivacaine [13]. In contrast, Fletcher et al. reported that no changes were observed in the success rate, onset time, or duration of analgesia by axillary fentanyl administration [14].

However these apparently controversial results may be due to either the difference in methodology or population variation. Although it is reported that opioid antinociception can be initiated by activation of peripheral opioid receptors [5, 25], it is not yet clear whether this is due to the fentanyl central effect or the peripheral removal of opioids into the blood circulation and further transport to the CNS. In none of the studies referred above, the intensity of pain and analgesic dose used after operation were not mentioned. However, it has been reported that the *μ*-opioid receptor activation leads to a sustained increase in glutamate synaptic effectiveness at the NMDA receptor level and is associated with central hypersensitivity to pain [16].

## 5. Conclusion

Based on the data found in our study, it could be concluded that addition of dexamethasone to lidocaine significantly prolonged the duration of analgesia compared with fentanyl/lidocaine mixture or lidocaine alone using axillary block in patients undergoing forearm fracture surgery. Furthermore, patients experienced pain at lower intensity following the operation compared to the other two groups. Moreover, the analgesic dose administered for the group that received dexamethasone after the surgery was significantly lower than that used in the other two groups. Considering the significant effect produced by the addition of dexamethasone to lidocaine, it is recommended that the combination of dexamethasone/lidocaine be used in axillary block. It is also suggested that similar comparisons by adding other agents such as ketamine, ephedrine, and clonidine to lidocaine be made in future studies while using both children and adult age groups, separately.

## Figures and Tables

**Table 1:** Demographic data for three study groups.

Groups	Group L (*n* = 19)	Group LF (*n* = 18)	Group LD (*n* = 21)	*P*
Age (years)	30.01 ± 5.47	31.28 ± 6.05	29.33 ± 6.65	0.59
Gender (F/M)	5/14	4/14	7/14	0.73

Values are presented as mean ± SD or number of patients. L: lidocaine; LD: combined lidocaine/dexamethasone; LF: combined lidocaine/fentanyl.

**Table 2:** Differences in analgesic consumption, VAS, onset, and duration of sensory and motor block of patients in three groups.

Groups	Group L (*n* = 19)	Group LD (*n* = 21)	Group LF (*n* = 18)	*P*
Onset time of sensory block (min)	1.9 ± 0.51	1.73 ± 0.51	1.72 ± 0.52	0.44
Duration of sensory block (min)	106 ± 18.03	206 ± 25.05	139 ± 22.89	<0.001
Onset time of motor block (min)	2.15 ± 0.6	2 ± 0.53	2 ± 0.43	0.68
Duration of motor block (min)	119 ± 20.84	225 ± 27.35	147 ± 24.62	<0.001
Total analgesic consumption (mg)	40.78 ± 14.93	25 ± 0.00	34.72 ± 12.54	<0.001
VAS (at time of return sensation)	7.42 ± 1.64	5.61 ± 1.28	6.27 ± 1.70	<0.001

L: lidocaine; LD: combined lidocaine/dexamethasone; LF: combined lidocaine/fentanyl; VAS: visual analog scale.

**Table 3:** Hemodynamic variables.

Group	Group L (*n* = 19)	Group LD (*n* = 21)	Group LF (*n* = 18)	*P*
SBP (during ABP)	130 ± 12.62	126 ± 12.70	131 ± 18.18	0.58
DBP (during ABP)	81.00 ± 8.06	77.85 ± 10.10	78.16 ± 8.59	0.49
HR (during ABP)	88.94 ± 11.08	79.76 ± 13.33	84.72 ± 10.41	0.6
SBP (5 minutes after ABP)	122 ± 10.91	123 ± 11.76	126 ± 15.97	0.66
DBP (5 minutes after ABP)	74.36 ± 9.85	73.76 ± 7.44	74.38 ± 6.83	0.96
HR (5 minutes after ABP)	77.26 ± 10.07	76.38 ± 11.91	78.72 ± 8.34	0.77
SBP (10 minutes after ABP)	120 ± 10.58	121 ± 11.62	122 ± 14.99	0.85
DBP (10 minutes after ABP)	72.15 ± 9.55	72.47 ± 7.52	73.27 ± 9.13	0.92
HR (10 minutes after ABP)	74.21 ± 7.16	73.90 ± 12.31	74.72 ± 946	0.96
SBP (15 minutes after ABP)	119 ± 11.44	117 ± 10.71	120 ± 13.39	0.59
DBP (15 minutes after ABP)	71.84 ± 9.96	70.09 ± 5.83	70.77 ± 7.39	0.78
HR (15 minutes after ABP)	73.89 ± 11.60	69.04 ± 10.25	69.72 ± 5.92	0.24
SBP (20 minutes after ABP)	118 ± 11.21	116 ± 10.58	119 ± 12.72	0.68
DBP (20 minutes after ABP)	69.73 ± 7.30	68.09 ± 6.86	69.44 ± 7.03	0.73
HR (20 minutes after ABP)	105 ± 14.4	67.61 ± 7.55	68.61 ± 5.63	0.27

Values are numbers or mean ± SD. L: lidocaine; LD: combined lidocaine/dexamethasone; LF: combined lidocaine/fentanyl; SBP: systolic blood pressure (mm Hg); DBP: diastolic blood pressure (mm hg); HR: heart rate (beats per minute); ABP: axillary block placement.
